# Emergence of Usutu virus in Southern Italy: phylogenetic profiling of potential introduction and dissemination routes

**DOI:** 10.3389/fvets.2026.1867579

**Published:** 2026-07-07

**Authors:** Alessia Pucciarelli, Lorena Cardillo, Gerardo Picazio, Maurizio Viscardi, Sara Cavaliere, Francesca Santomartino, Marita Georgia Riccardi, Alessia Napolitano, Maria Lella, Giovanna Fusco, Nicola D'Alessio, Giorgio Galiero, Claudio de Martinis

**Affiliations:** 1 Department of Animal Health, Istituto Zooprofilattico Sperimentale del Mezzogiorno, Naples, Italy; 2 Departmental Unit of Genetics, Bioinformatics and Biobank, Istituto Zooprofilattico Sperimentale del Mezzogiorno, Naples, Italy; 3 Istituto Zooprofilattico Sperimentale del Mezzogiorno, Caserta, Italy

**Keywords:** lineage Europe 2, molecular epidemiology, Sanger sequencing, surveillance, Usutu virus

## Abstract

Usutu virus (USUV) is an emerging mosquito-borne flavivirus closely related to West Nile virus (WNV), sharing ecological niches and transmission cycles involving *Culex* mosquitoes and wild birds. In Italy, USUV was first detected in 1996 and has become endemic in northern regions, whereas, to date, no cases have been reported in southern Italy. In August 2025, a yellow-legged gull (*Larus michahellis*) from the provinces of Salerno for passive surveillance and a Eurasian jay (*Garrulus glandarius*) during active surveillance from Caserta (southern Italy), were examined. Heart, spleen, kidney, and brain tissues were collected for virological investigation. Real-time RT-PCR assays targeting West Nile virus and Usutu virus were performed and USUV RNA was detected in both avian specimens, while samples tested negative for WNV. Next, entomological surveillance was conducted on both sites and a mosquito pool collected near the yellow-legged gull carcass tested positive. Positive samples were subjected to Sanger sequencing of the NS5 gene. BLAST and phylogenetic analyses revealed strain belonged to lineage Europe 2, sub-group 2-A showing 99.6% nucleotide identity with northern Italian strains circulating between 2018 and 2023 and 99.8% identity with northern European strains circulating between 2016 and 2024. These findings suggest two possible scenarios: local southward spread of an autochthonous Italian strain or a recent introduction from other European countries mediated by migratory wild birds. This study provides the first molecular evidence of USUV circulation in southern Italy and underscores the critical role of molecular epidemiology and phylogenetic analysis in tracing routes of pathogen introduction and spread.

## 1 Introduction

Usutu virus (USUV) is a mosquito-borne Flavivirus belonging to the Japanese encephalitis virus complex, which is closely related to West Nile virus (WNV). These two neurotropic mosquito-borne viruses are maintained through similar enzootic transmission cycles involving ornithophilic mosquitoes and avian amplifying hosts, including both resident and migratory birds (1). USUV was first isolated in 1959 from *Culex neavei* in South Africa (2) and, from a genetic perspective, is classified into multiple lineages according to their putative geographical origin, namely Africa 1–3 and Europe 1–4. More recently, a novel lineage, designated Europe 5, has been proposed based on partial NS5 sequencing (3).

Natural infections caused by USUV have also been documented by serological and epidemiological evidence in a range of incidental mammalian hosts, including humans, bats, equines, dogs, and ruminants. In humans and horses, however, infection is generally considered an epidemiological dead end (1, 4). Attention has recently been directed toward USUV infection in humans because of its increasing circulation in Europe and its potential neuroinvasive capacity. Human USUV infections are frequently asymptomatic and are often identified retrospectively through serological investigations or blood donor screening. When symptomatic, infection may present with mild and nonspecific clinical manifestations, including fever, headache, rash, nuchal rigidity, tremor, and hyperreflexia, with headache, fever, and nuchal rigidity representing the most commonly reported symptoms (1). Severe neurological disease has been mainly described in immunocompromised and/or elderly individuals, presenting with febrile illness, meningitis, or meningoencephalitis (1, 5).

The first detection of USUV in Europe dates to 1996 in Italy, when a marked mortality event among wild birds, particularly Eurasian blackbirds (*Turdus merula*), was reported in Tuscany. From 2001 onwards, USUV expanded geographically and was identified in several European countries, including Austria, Hungary, Switzerland, and Germany (6). Since then, the virus has become established across multiple western, southern, and central European regions, where it has been documented in humans, a range of vertebrate hosts, and mosquito vectors (5, 7).

In Italy, since its introduction, USUV has spread from Tuscany to the Trentino region in 2005 (8) when it was evidenced in sentinel chickens, in Emilia Romagna in 2007 (9) and in Lombardy between 2006–2008 (10, 11). Currently, the virus is reported in several regions, mostly located in the northern/central territories, suggesting continuous viral circulation and overwintering capability (12, 13). Furthermore, it has been evidenced the circulation of viruses belonging to four different Europe lineages (EU1, EU2, EU3 and EU4), with Europe 2 in the north-eastern and central regions (Emilia-Romagna, Veneto, FVG, Marche, Tuscany, Lazio Regions), and Europe 4 in the north-western and central regions (Lombardy, Piedmont, Marche) (12, 14). Moreover, an evolutionary diversification within the EU2 lineage has been reported, resulting in two distinct sub-lineages (EU2-A and EU2-B), indicating a local viral evolution. While EU2-A appears to be more widespread across northern and central Italy, EU2-B remained mainly restricted to the Veneto region (14).

In southern Italy, information on circulating USUV strains remains limited, indeed, in recent years, USUV was detected only in Sicily, where a wild bird tested positive in the Palermo province in 2021 (15), with no additional detections reported from other southern regions, including Campania.

In this context, Italian regions are risk-based classified into categories, according to the National Surveillance Plan for Arbovirosis (PNA 2020–2025), which establishes an integrated surveillance framework for WNV and USUV, targeting their circulation in resident avifauna, wild bird mortality, mosquito vectors, equids, and humans. Therefore, three categories are identified: (i) high-risk areas, where viral circulation is ongoing or has occurred within the previous 5 years; (ii) low-risk areas, where sporadic circulation has been documented or eco-climatic conditions may favor virus emergence; and (iii) minimal-risk areas, where the virus has never been detected and environmental conditions are considered unsuitable for circulation (16). On these bases, before 2020, the Campania region was classified as low-risk area, as neither WNV or USUV had been reported. However, since 2020 the epidemiological scenario has rapidly evolved with the emergence and subsequent widespread circulation of WNV, initially Lineage 1 (16, 17) and later Lineage 2, leading to endemic establishment (18, 19). Consequently, Campania is currently considered a high-risk region for WNV, while USUV has not yet been identified.

The present study reports the first molecular characterization of USUV detected in a yellow-legged gull (*Larus michahellis*), a Eurasian jay (*Garrulus glandarius*) and a *Culex pipiens* mosquito pool, in the Campania region. This finding contributes to the molecular epidemiological knowledge of USUV circulation in Italy and provides insight into its persistence and potential ecological drivers.

## 2 Material and methods

### 2.1 Sample collection

During passive surveillance activities conducted by the Local Veterinary Services in accordance with the Italian National regulation for arbovirosis (PNA 2020–2025) and Regional plan for prevention, surveillance, and response to arbovirosis (20), in August 2025 a yellow-legged gull (*Larus michahellis*) was found dead in the municipality of Eboli (Salerno province, Campania region, southern Italy). In the same period, within the framework of the active surveillance program established by the same national regulation and in compliance with the depopulation measures therein prescribed, a Eurasian jay (*Garrulus glandarius*) was culled in the municipality of Alvignano (Caserta province, Campania region, southern Italy). Both carcasses were conferred to the Istituto Zooprofilattico Sperimentale del Mezzogiorno (Portici, Naples, southern Italy) and examined at the Unit of Wildlife, where complete necropsy was performed for the determination of the cause of death. Samples of brain, spleen, heart, and kidney were collected from each specimen for subsequent laboratory analyses. Additionally, a mosquito pool, collected near the yellow-legged gull in October 2025 using a CO_2_-baited CDC light traps as part of the entomological surveillance plan, was included in the virological investigation. The collected mosquitoes were identified using morphological criteria under stereomicroscopy and taxonomic identification was performed using validated identification keys implemented in MosKeyTool. Mosquitoes were divided into two pools of 15 specimens each.

### 2.2 Homogenization and nucleic acids extraction

Each pool was placed in a 2 mL microcentrifuge tube (Eppendorf, Hamburg, Germany) containing 1.5 mL of PBS and processed using sterile silica sand with a particle diameter of 0.1 mm (Benchmark Silica Glass Bulk Beads, Sigma-Aldrich, St. Louis, USA). Moreover 2 g of each tissue sample were suspended in 2 mL of sterile PBS in 2 mL tubes (Eppendorf). All samples were homogenized with a 4.8 mm stainless steel bead using a TissueLyser (Qiagen, Hilden, Germany) at 30 Hz for 5 min to promote mechanical lysis and then centrifuged at 2,000*g* for 5 min (Eppendorf). From the clarified supernatant, 200 μL aliquots were collected for nucleic acid extraction and purification using KingFisher Flex instrument (Thermo Fisher Scientific, Waltham, MA, USA) with MVP_2Wash_200_Flex protocol, following the manufacturer's instructions. The nucleic acids were eluted in 60 μL and stored at −80 °C until analysis. Negative extraction controls were included in all procedures.

### 2.3 Molecular analyses and sequencing

Eluates were analyzed by one-step real-time RT-PCR for the simultaneous detection of West Nile Virus lineage 1 and 2 (21), and USUV (22) using the protocols described in Table 1.

**Table 1:** Primer sets, probes and thermal profile used for the detection of West Nile Virus and USUTU virus.

Virus	Primer and probe sequence (5^′^to 3^′^)	Thermal profile				References
		Step	Temperature (°C)	Time	*n* cycles	
West Nile virus	WN-LCV-F1 (0.4 μM) 5′-GTGATCCATGTAAGCCCTCAGAA-3′	Reverse Transcription (RT)	50°	30 min	1 cycle	Del Amo et al. (21)
	WN-LCV-R1 (0.4 μM) 5′-GTCTGACATTGGGCTTTGAAGTTA-3′	Enzymatic activation	95°	15 min	1 cycle	
	WN-LCV-S1 (0.2 μM) 5′-FAM-AGGACCCCACATGTT-3′-MGB	Denaturation	95°	15 s	45 cycles	
	WN-LCV-S2 (0.2 μM) 5′-VIC-AGGACCCCACGTGCT-3′-MGB	Annealing/Exstension	60°	60 s		
USUV	USU-F (0.9 μM) 5′-AAAAATGTACGCGGATGACACA-3′	Reverse Transcription (RT)	48°	3 min	1 cycle	Cavrini et al. (22)
	USU-R (0.9 μM) 5′-TTTGGCCTCGTTGTCAAGATC-3′	Enzymatic activation	95°	10 min	1 cycle	
	USU-P (0.25 μM) 5′-FAM-CGGCTGGGACACCCGGATAACC-TAMRA-3′ ' BHQ1	Denaturation	95°	15 s	40 cycles	
		Annealing/Exstension	60°	60 s		

Briefly, West Nile Virus Lineages 1 and 2 reaction was performed in 25-μL using QuantiTect Probe RT-PCR Kit (Qiagen) which included 1 × final concentration of QuantiTect RT-PCR Master Mix, 0.4 μM for each primer and 0.2 μM for each probe, along with 1 × Xeno Liz Primer and Probe Mix, 5 μL of template, and RNAse/DNase free water to reach the final volume. USUV reactions were performed in 25 μL final volume with TaqMan Fast Virus 1-Step Master Mix (Thermo Fisher Scientific), containing 1 × Master Mix, 0.9 μM of each primer, 0.25 μM probe, 1 × VetMAX Xeno Internal Positive Control—VIC Assay (Applied Biosystems, Thermo Fisher Scientific), and 5 μL of RNA template. Amplification targeted a 73 bp fragment of the NS5 gene. Reactions were run on a QuantStudio5 instrument (Applied Biosystems, Thermo Fisher Scientific) in the presence of positive and negative controls (16). Positive samples were further analyzed using endpoint PCR assay with USUV-specific diagnostic primers already reported by Hubálek et al. (23), Usna129f, 5′-AGGACCATTGGTTAGGAAGA-3′ and Usna663r, 5′-GGCTTGACAACACAATCATC-3′, which amplify a fragment of approximately 535 bp, with SuperScript IV One-Step RT-PCR System (Thermo Fisher Scientific) following the manufacturer's instructions. Reverse transcription was performed at 50 °C for 30 min, followed by a denaturation step at 98 °C for 2 min. Thereafter the cDNA was amplified in 40 cycles (heat denaturation at 94 °C for 40 s, primer annealing at 57 °C for 50 s, and DNA extension at 72 °C for 1 min), and the reaction was completed by a final extension for 7 min at 72 °C. Next, 1 μL of the amplification products was analyzed by capillary electrophoresis with Agilent TapeStation 4150 system (Agilent Technologies, Santa Clara, CA, US) and D1000 ScreenTape (Agilent Technologies). Amplicons were then purified by QIAquick PCR Purification kit (Qiagen) and sequenced in both directions using the same endpoint PCR primers along with BigDye Terminator v.1.1 Cycle Sequencing Kit (Applied Biosystems, Thermo Fisher Scientific) according to the manufacturer's instructions on T100 Thermal Cycler (Bio-Rad Laboratories, Segrate, Milan, Italy). A final purification was performed using DyeEx 2.0 Spin Kit (Qiagen) followed by denaturation with formamide at 95 °C for 5 min and then sequenced by capillary electrophoresis on ABI-Prism 3500 Genetic Analyzer (Applied Biosystems, Thermo Fisher Scientific). Sequences were analyzed with the SeqScanner 2.0 software (Applied Biosystems, Thermo Fisher Scientific) for quality check.

A molecular genotyping analysis was subsequently conducted on the collected mosquitoes, to detect allelic variants associated with resistance to pyrethroid insecticides. The allele-specific PCR assay allows to identify the *kdr* mutation leucine to phenylalanine at position 1014 of the voltage-sensitive sodium channel gene (*vssc gene*). In detail, two PCR reactions were run in parallel as described by Martinez-Torres et al. (24). In one reaction, in a total volume of 25 μL, 0.2 μM of Cdg1, Cdg2 primers and 0.3 μM of Cdg3 reverse primer were combined. In the second reaction, Cdg3 was replaced by 0.3 μM of Cdg4 reverse primer. Cdg1 and Cdg2 are universal primers binding in the genomic region flanking the mutation, generating an outer aspecific amplicon of 481–510 bp. Cdg 3 and Cdg4 primers are allele specific. The product size for the allele-specific (wildtype) amplicons is 354–383 bp.

### 2.4 Phylogenetic analysis

Consensus sequences were obtained by the Geneious software version 9.1.8 (Biomatters Ltd., Auckland, New Zealand) and compared with those available in the GenBank database by the Basic Local Alignment Search Tool (BLAST; http://blast.ncbi.nlm.nih.gov/Blast.cgi).

For lineage assignment, USUV reference sequences were retrieved from GenBank using BLAST searches, including both partial and complete genomes. Only the NS5 gene region overlapping with the newly generated sequences was retained for subsequent analyses. A total of 123 USUV reference sequences, representative of all currently recognized lineages, were retrieved from GenBank and included in the final dataset. Sequence selection was performed to ensure geographical and lineage representativeness, with particular focus on strains previously detected in Italy and other European countries. Within the EU2-A lineage, sequences showing the highest nucleotide identity with the strain identified in this study were preferentially included, together with representative sequences displaying lower nucleotide identity, to provide a robust phylogenetic lineage assignment.

Sequence alignment was performed using MUSCLE as implemented in MEGA version 12. The best-fitting nucleotide substitution model was identified using the “Find Best DNA/Protein Models” option in MEGA, resulting in the selection of the Tamura–Nei (TN93) model. Phylogenetic relationships were inferred from the NS5 gene using the maximum likelihood method under the TN93 nucleotide substitution model. Rate heterogeneity among sites was modeled using a discrete gamma distribution with five categories (+G), with no invariant sites (+I = 0). Statistical support for the inferred topology was evaluated using standard bootstrap analysis with 1,000 replicates. All evolutionary analyses were conducted in MEGA version 12 using up to eight parallel computing threads. The resulting phylogenetic tree was exported in Newick format and visualized and annotated using Interactive Tree Of Life web tool (iTOL, https://itol.embl.de).

## 3 Results

Among the tested organs, USUV positivity was detected in the kidney (cycle threshold [Ct] value: 28.5) and spleen (Ct: 25.2) samples from the yellow-legged gull, and in the spleen sample (Ct > 35) from the Eurasian jay. In addition, the *Culex pipiens* mosquito pool tested positive for USUV by real-time RT-PCR analysis (Ct: 19). All samples tested negative for WNV. Results were subsequently confirmed by the National Reference Centre for Exotic Animal Diseases (CESME, Istituto Zooprofilattico Abruzzo e Molise, Teramo, Italy).

Samples that tested positive underwent Sanger sequencing targeting the NS5 region, yielding high-quality amplicons. Bidirectional chromatogram analysis of the purified products enabled the generation of a 538 bp consensus sequence from the yellow-legged gull spleen sample and a 496 bp consensus sequence from the mosquito pool sample. The gull-derived consensus sequence was slightly longer than the expected amplicon size (~535 bp), likely due to minor terminal length variation relative to the reference sequence and inclusion of complete end regions during sequence assembly. In contrast, inconclusive sequencing results were obtained for the Eurasian jay spleen sample, probably because of the high Ct value.

The sequences obtained from the spleen of the yellow-legged gull and from the mosquito pool were deposited in the NCBI GenBank database under accession numbers PX443590 and PX872760, respectively. BLAST analysis demonstrated a 99.80% nucleotide identity between the two sequences, indicating a very high level of genetic similarity.

Furthermore, comparative analysis with previously reported USUV strains revealed high degree of genomic conservation among the analyzed sequences and strains circulating both in Italy and Central Europe in recent years. Specifically, the sequences shared 99.63% nucleotide homology with Italian strains detected in 2018 in the Veneto region from a mosquito pool (GenBank: MW164731.1) and in 2023 in the Umbria region from a blackbird (*Turdus merula*; GenBank: PP104388.1).

Moreover, a 99.81% nucleotide identity was observed with Northern-central European strains, including isolates obtained in 2017 in Hungary from *Turdus merula* (GenBank: MF063046.1), in 2016 in the Czech Republic from a *Culex modestus* pool (GenBank: MN419895.1), and in 2024 in Germany from a human case (GenBank: PX068495). Although these findings indicate a close genetic relationship with strains circulating in both Italy and other European countries, the limited divergence observed across the analyzed 535-bp NS5 fragment does not provide sufficient phylogenetic resolution to confidently distinguish between alternative viral introduction scenarios or transmission routes.

**Figure 1:**
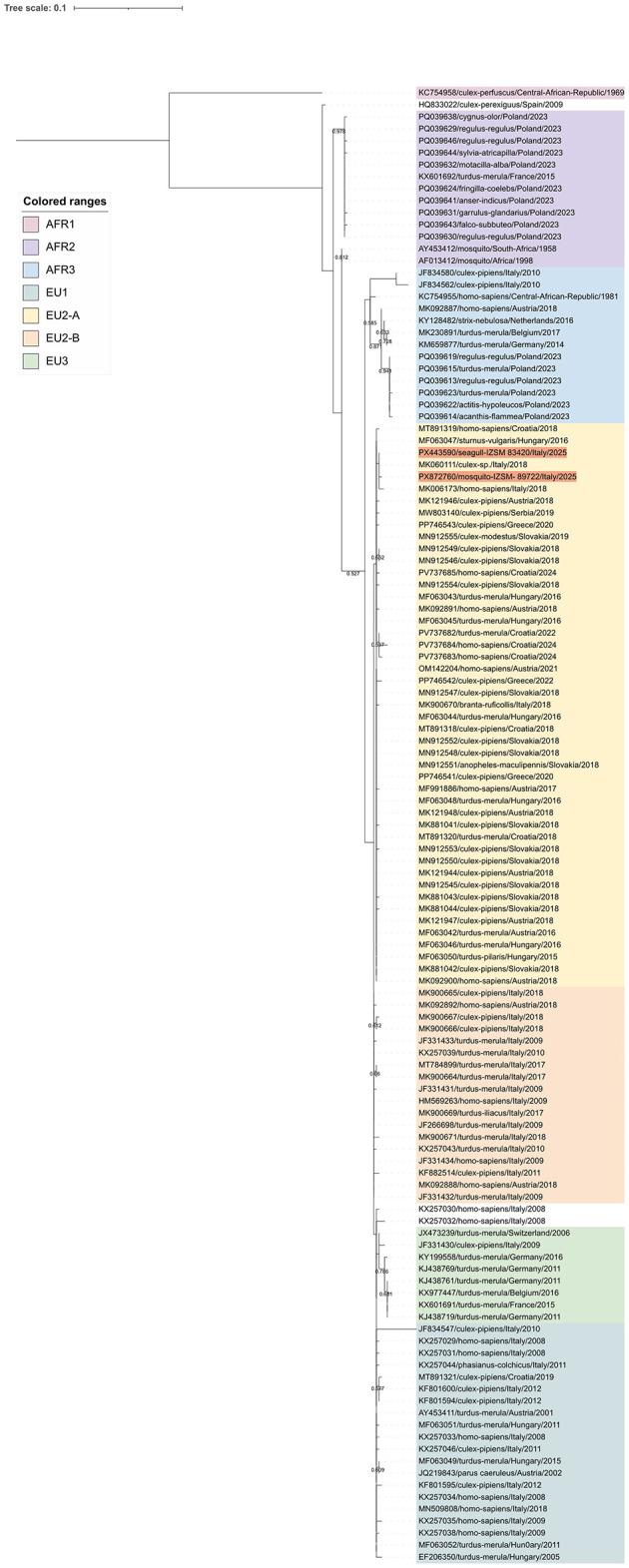
Phylogenetic tree of Usutu virus sequences inferred from the NS5 gene using the maximum likelihood method under TN93 nucleotide substitution model.

Phylogenetic analysis based on the NS5 gene showed that the two newly generated USUV sequences obtained in the present study from the Campania region, clustered within the European EU2-A lineage. Both sequences grouped together with the previously reported European strains, forming a well-defined EU2-A clade. The overall tree topology was consistent with the established phylogenetic structure of USUV, clearly separating African and European lineages. The bootstrap support values confirm the separation of the main USUV lineages, as well as the placement of the Campania sequences within the EU2-A lineage. However, bootstrap support for relationships at the level of more specific subclades was lower, likely due to the relatively short length of the 534-bp NS5 fragment analyzed (Figure 1).

Finally, molecular genotyping of the *C. pipiens* sample for allelic variants associated with pyrethroid resistance identified a heterozygous L1014F (L/F) genotype at the voltage-gated sodium channel (*vgsc*) locus (24). This finding indicates the presence of the resistance-associated (1014F) allele within the population, although no homozygous resistant (F/F) individuals were detected. The heterozygous condition is typically associated with reduced susceptibility or an intermediate resistance phenotype compared with fully susceptible (L/L) mosquitoes, suggesting ongoing selective pressure by pyrethroid insecticides without evidence of fixation of the resistant genotype.

## 4 Discussion

The present study provides the first molecular evidence of USUV circulation in a yellow-legged gull (*Larus michahellis*), a Eurasian jay (*Garrulus glandarius*), and *Culex pipiens* mosquitoes in the Campania region, detected within the framework of passive and active surveillance activities implemented under the Italian National Surveillance Plan for Arbovirosis (PNA 2020–2025) and Regional plan for prevention, surveillance, and response to arbovirosis (20). In recent years, climate and environmental changes, particularly rising temperatures, modified precipitation patterns, and expanding urban–periurban interfaces, have promoted the proliferation and seasonal persistence of mosquitoes such as *Culex pipiens*, which is known to be a competent vector for USUV (25, 26). These ecological drivers facilitate viral amplification and contribute to the progressive geographic spread of mosquito-borne flaviviruses across the Mediterranean basin (27). Furthermore, climate change has been associated with significant alterations in avian migratory behavior, including shifts in migratory routes, changes in phenology, and prolonged stopover or overwintering periods in previously transitional areas. These ecological modifications can substantially influence the epidemiology of vector-borne infectious diseases carried by migratory birds. Consequently, such dynamics may promote the introduction, establishment, and subsequent spread of pathogens into newly colonized territories, including areas previously considered free from specific arboviral diseases (27, 28). Vector-borne pathogens such as WNV and USUV are typically introduced into new territories through infected migratory birds. Once introduced, local mosquito populations sustain transmission by infecting resident avian species, thereby enabling viral establishment and eventual endemicity (29). Notably, the avian species identified in the present study are predominantly resident. The yellow-legged gull is widely distributed along Mediterranean coastal ecosystems, while the Eurasian jay is considered a target species due to its documented susceptibility to flavivirus infection and its role in viral amplification dynamics. Therefore, the detection of USUV in these resident hosts strongly suggests active local circulation rather than a transient introduction event (12). The concurrent detection of USUV in resident birds and *Culex pipiens* mosquitoes may indicates that the virus is maintained within the regional ecosystem. Although initial viral introduction may occur via migratory birds along established flyways, the evidence presented here could support local transmission in the Campania region. However, the 2-month interval between avian and mosquito sampling represents a limitation for the epidemiological interpretation of these findings. Considering the seasonal dynamics and short lifespan of *Culex pipiens* mosquitoes, the detected viruses may not reflect the same transmission event. Therefore, the increasing frequency of bird mortality events associated with USUV infection highlights the value of integrated avifaunal and entomological surveillance as an early warning system for arboviral circulation with potential zoonotic implications (7). From a surveillance perspective, our findings reinforce the importance of an integrated arbovirus monitoring targeting simultaneously USUV and WNV, given their overlapping ecological niches, shared *Culex pipiens* vector, and common avian hosts (13). Such a coordinated approach strengthens early detection capacity and supports a One Health strategy linking wildlife health, vector ecology, and public health preparedness. Furthermore, genomic sequencing plays a pivotal role in modern disease surveillance. Molecular characterization of circulating strains allows fine-scale phylogenetic reconstruction, enabling molecular tracing of viral introductions, identification of potential geographic sources, and monitoring of spatial dissemination patterns (30). In this context, the identification of lineage Europe 2 in this study is consistent with the predominant genotype circulating in Italy and other European countries, suggesting long-term adaptation to endemic host-vector systems (13, 27). Within this lineage, two sub-lineages, EU2-A and EU2-B, have been described, with different distribution (14), where EU2-A is more widely distributed in northern and central Italy, as well as in Austria and Hungary (14), also corroborated by our findings. The phylogenetic analysis performed in the present paper revealed high nucleotide identity between the strains detected in this study and those previously reported in northern and central Italy. However, the limited divergence observed in the 535-bp NS5 fragment analyzed provides limited phylogenetic resolution to distinguish with certainty between a possible southward spread within Italy and independent introduction events from other European regions. Therefore, although the sequences obtained are consistent with circulation within the EU2-A lineage, additional whole-genome sequencing data and expanded surveillance activities will be necessary to robustly reconstruct viral spread pathways and evolutionary dynamics. However, tdefined EU2-A clade. The overall tree topology was consistent with the established phylogenetichis genetic relatedness supports the hypothesis of progressive viral dispersion along major ecological corridors, including the Tyrrhenian coastal axis and the Apennine ridge, likely mediated by both migratory and resident avifauna (7, 12). Nevertheless, even higher nucleotide identity (99.81%) was observed with strains identified in northern-central European strains, such as Hungary, the Czech Republic and Germany, opening a second scenario of a possible novel introduction from these areas, where EU2-A has circulated Overall, the data support two alternative hypotheses: (i) the circulation and progressive spread of an autochthonous Italian strain from northern toward central and southern Italy, or (ii) a novel introduction from northern-central Europe followed by local circulation in Italy. However, the partial NS5 gene fragment analyzed in this study does not provide sufficient phylogenetic resolution to clearly discriminate between these two scenarios, although NS5 has already been reported as a well-established and informative molecular marker for USUV lineage classification (31). Additional whole-genome sequencing data and expanded surveillance activities will be necessary to robustly reconstruct viral dissemination routes and evolutionary dynamics.

In conclusion, the results of this study underscore the strategic importance of integrated surveillance systems combining avifaunal monitoring and entomological investigations to provide early warning of vector-borne disease circulation. The simultaneous assessment of wild birds and mosquito populations enables timely detection of enzootic amplification, supports targeted vector control interventions, and contributes to optimized disease management at the territorial level. Importantly, because humans represent incidental dead-end hosts for USUV and WNV, early evidence of viral activity in wildlife and vectors can provide predictive information on the potential occurrence of human cases, thereby increasing the level of epidemiological alert and improving public health responsiveness.

Continuous genomic surveillance is therefore essential to detect evolutionary dynamics, assess viral adaptation processes, and reconstruct transmission pathways across ecological corridors (30). The integration of genomic data with ecological and epidemiological information represents a critical tool for understanding virus emergence, guiding risk assessment, and strengthening preparedness strategies in Mediterranean ecosystems characterized by high biodiversity and intense vector activity.

## Data Availability

The datasets presented in this study can be found in online repositories. The names of the repository/repositories and accession number(s) can be found in the article/supplementary material.
